# Carbon Contamination During Ion Irradiation - Accurate Detection and Characterization of its Effect on Microstructure of Ferritic/Martensitic Steels

**DOI:** 10.1038/s41598-017-15669-y

**Published:** 2017-11-17

**Authors:** Jing Wang, Mychailo B. Toloczko, Karen Kruska, Daniel K. Schreiber, Danny J. Edwards, Zihua Zhu, Jiandong Zhang

**Affiliations:** Pacific Northwest National Laboratory, Richland, WA 99354 USA

## Abstract

Accelerator-based ion beam irradiation techniques have been used to study radiation effects in materials for decades. Although carbon contamination induced by ion beams in target materials is a well-known issue in some material systems, it has not been fully characterized nor quantified for studies in ferritic/martensitic (F/M) steels that are candidate materials for applications such as core structural components in advanced nuclear reactors. It is an especially important issue for this class of material because of the strong effect of carbon level on precipitate formation. In this paper, the ability to quantify carbon contamination using three common techniques, namely time-of-flight secondary ion mass spectroscopy (ToF-SIMS), atom probe tomography (APT), and transmission electron microscopy (TEM) is compared. Their effectiveness and shortcomings in determining carbon contamination are presented and discussed. The corresponding microstructural changes related to carbon contamination in ion irradiated F/M steels are also presented and briefly discussed.

## Introduction

Accelerator-based self-ion irradiation has been used to study radiation effects in solid materials for decades^1–5^. Recently, there has been renewed interest in using it as a surrogate technique to investigate neutron damage for a variety of reasons that include reduced availability of neutron irradiation facilities, its capability to achieve high damage dose within a short timeframe, lack of activation of the specimens, and low cost. To fully exploit the advantages of ion irradiation, correlations and differences among ion-induced and neutron-induced damage must be understood. A number of neutron-atypical factors have been identified for ion irradiation, such as high dose rate, irradiation beam scanning mode, surface sputtering, and injected ions^6–10^. Besides these identified effects, recent studies using ion irradiations of ferritic/martensitic (F/M) steels have revealed another neutron atypical variable, carbon contamination, that appears to have been neglected for ion irradiation studies of reactor structural materials^11^.

Carbon contamination has been a well-known issue for ion beam usage and analysis since the 1970s^12–18^, but the potential for it to cause issues in studying radiation effects in reactor structural materials does not appear to have been recognized. It is an important issue for F/M steels and austenitic steels where carbon has a strong effect on carbide formation that can effect microstructural development and response to irradiation such as swelling behavior^19^. To the best of our knowledge, little effort has been put into quantification of carbon contamination in ion irradiated F/M steels. The source of carbon contamination has been attributed to free hydrocarbons, CO, or CO_2_ molecules in the vacuum system. Reducing the concentration of carbon-containing molecules with a liquid nitrogen trap in the proximity of the target specimens has proven to be effective in the past. An alternative technique that utilizes a magnetic beam deflector together with a liquid nitrogen cold trap to minimize carbon contamination has been proposed by Shao *et al*.^11^. Although this study is focused on carbon contamination, other contaminants, like oxygen and nitrogen, may also be present.

Because the extent of carbon contamination may vary from accelerator to accelerator, and because this is an emerging issue without a universal solution that guarantees no carbon contamination, it is important for anyone performing ion irradiations on steels or other materials strongly sensitive to carbon to assess whether carbon contamination has occurred in their specimens. This paper seeks to provide practical advice for efficient, reliable quantification of carbon contamination in ion irradiated materials. The details of materials, the ion irradiation experiment, and characterization techniques are presented in the Materials and Experimental section. Three investigation tools are compared and contrasted, namely time-of-flight secondary ion mass spectroscopy (ToF-SIMS), atom probe tomography (APT) and transmission electron microscopy (TEM). APT and TEM are popular microstructural characterization techniques for structural materials while SIMS is routinely used for probing ion implantation profiles in the semiconductor industry. The quantification from each technique will be discussed as well as their strengths and limitations. Microstructures of ion irradiated HT-9 characterized by APT and TEM will be presented to reinforce carbon contamination’s influence on microstructure during ion irradiation. Possible effects of carbon contamination on microstructural evolution are also discussed.

## Results

**Figure 1:**
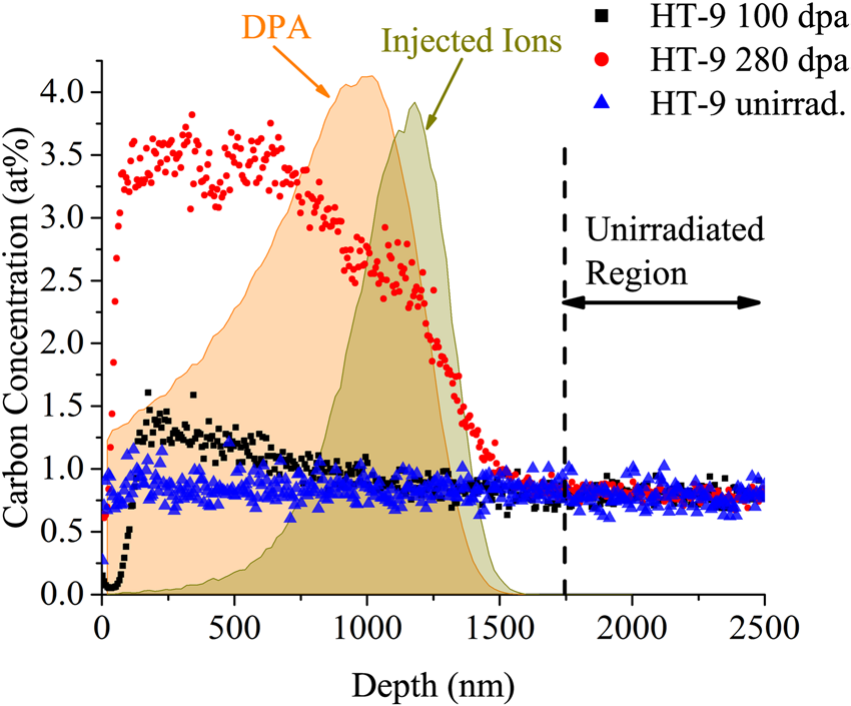
Depth profile of carbon concentration in unirradiated HT-9 and in HT-9 irradiated to 100 and to 280 dpa at 400 °C.

Figure 1 shows depth profiling of the carbon concentration as determined by ToF-SIMS in unirradiated HT-9 and HT-9 irradiated to either 100 dpa or 280 dpa. Injected ion profiles and dpa calculated by Stopping Range of Ions in Matter (SRIM) are scaled and overlaid in the same figure. The carbon concentration profile in the unirradiated HT-9 specimen is quite flat. Within ~100 nm of the surface of the irradiated HT-9 specimens, the carbon signal is low due to the existence of a surface oxide layer (data are not shown here for brevity) that may exert perturbations on secondary ion yield in SIMS. Beyond that region, it can be seen that major carbon contamination exists in the irradiated regions of both ion irradiated HT-9 specimens. The peak carbon concentrations are ~1.4 at% and ~3.5 at% in 100 and 280 dpa specimens, respectively, several factors higher than the nominal value of 0.8 at% in unirradiated HT-9.

**Figure 2:**
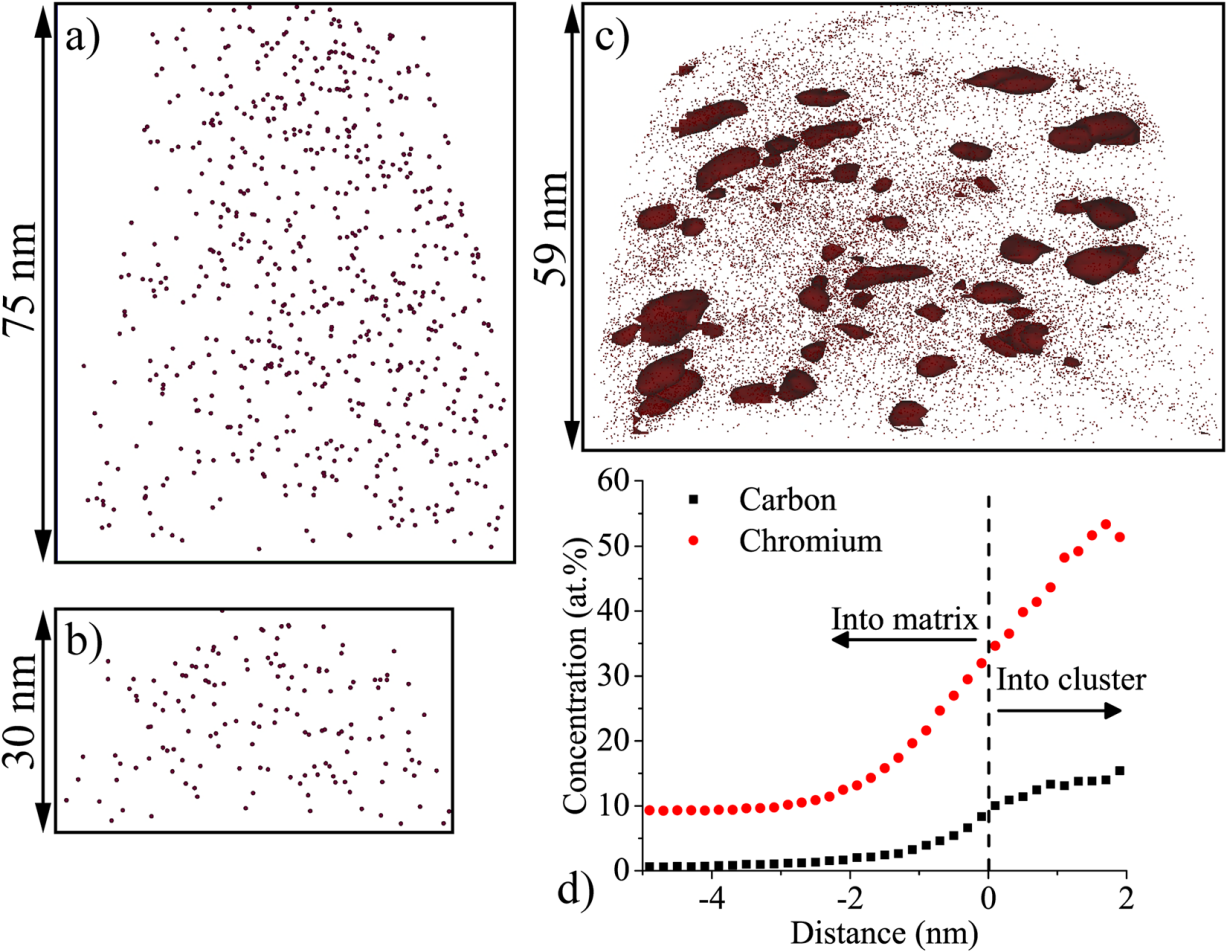
(**a**) and (**b**) Carbon ion maps in APT datasets extracted from unirradiated material. Scales of the datasets are labeled beside arrows; (**c**) carbon ion map and carbon clusters in HT-9 irradiated to 100 dpa at 400 °C; carbon clusters were defined by 6.8 at% C isoconcentration surfaces; (**d**) proxigram of Cr and C for those isoconcentration surfaces (interface located at 0 nm).

While SIMS is good at providing composition that is representative of the bulk, APT measures the spatial distribution of elements with relatively high accuracy, allowing visualization of the spatial details of the carbon distribution. Figures 2a and 2b show the carbon ion maps of two reconstructed APT tips from an unirradiated HT-9 specimen. No grain boundaries or grain boundary (GB) carbides were intersected, thus giving a view of the matrix carbon. The carbon ions are randomly distributed through the entire analysis volumes. Measured carbon concentrations are 0.017 and 0.024 at% in tips unirrad-1 and unirrad-2, respectively, as listed in Table 1. It is not unexpected that these numbers are much lower than the nominal value, ~0.8 at%, because most carbon is partitioned into GB carbides in normalized and tempered ferritic/martensitic alloys, and carbon solubility in BCC Fe-based steels is very low^20^.

**Table 1:** Chemical composition (at%) of unirradiated and ion irradiated materials measured by APT. Compositions were computed using “peak decomposition” for mass spectra in Integrated Visualization and Analysis Software (IVAS).

Specimens	Depth (nm)	Fe	Cr	C	Si	Mn	W	V	Mo	Ni
HT-9, Unirrad.	Unirrad-1	86.68	10.93	0.017	0.52	0.50	0.10	0.27	0.44	0.54
	Unirrad-2	86.66	10.99	0.024	0.54	0.47	0.10	0.29	0.44	0.49
HT-9, 100 dpa, 400 °C	0–60	85.39	11.12	1.10	0.52	0.47	0.10	0.29	0.45	0.56
HT-9, 280 dpa, 400 °C	300–440	83.63	10.58	3.33	0.53	0.47	0.10	0.28	0.51	0.57
	500–600	85.22 (79.96)†	9.27 (12.71)	2.97 (4.65)	0.51	0.57	0.11	0.24	0.55	0.66 (0.70)
	700–800	84.38	10.28	2.92	0.52	0.45	0.10	0.28	0.48	0.59
	1000–1250	85.81	9.92	2.03	0.50	0.44	0.10	0.26	0.43	0.51

^†^Values in parenthesis represent concentrations including the two GB carbides in this APT tip.

The APT specimen shown in Fig. 2c) was extracted near the surface of the HT-9 specimen irradiated to 100 dpa at 400 °C. In this dataset, a very high number density of small carbon-rich clusters with diameters of several nanometers were observed. The radial concentration profiles (proxigrams) of Cr and C computed around the detected clusters are plotted in Fig. 2d). The measured Cr/C ratio near the center of the cluster is ~3.79, which corresponds to the ratio in M_23_C_6_ (3.83). While it is tempting to conclude that the clusters are M_23_C_6_, it will be shown by TEM analysis that they are likely to be Cr and C enriched α clusters. The carbon concentration of the entire tip is ~1.1 at% (see Table 1). Based on APT analysis alone, it is not possible to identify this precipitate as carbon contamination. While it could be attributable to carbon contamination, it is also possible that it could be due to dissolution of preexisting GB carbides followed by redistribution and reprecipitation of carbon. To determine whether it is due to preexisting carbide dissolution, it would be necessary to perform wider area examinations either by scanning electron microscopy (SEM) or TEM.

**Figure 3:**
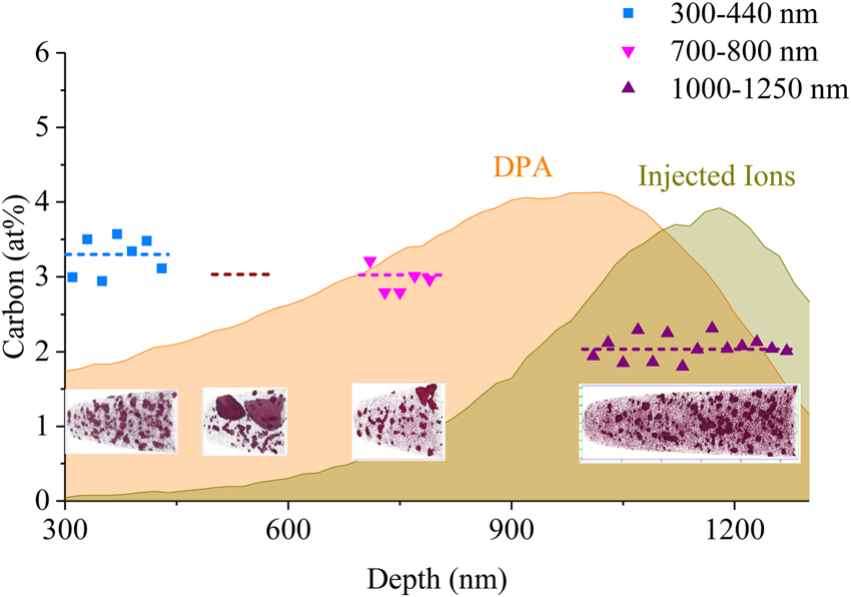
Depth-dependent carbon concentration measured by APT in HT-9 irradiated to 280 peak dpa at 400 °C, superimposed with SRIM-calculated dpa and injected ion profiles. Dashed lines represent average carbon concentrations of the entire APT needles. For the APT needle taken from a depth of 500–600 nm, the dashed line is calculated using a volume that does not contain GB carbides, and individual values from 20 nm thick slices were not meaningful for this APT needle because of the effect of the GB carbides on the slices.

Figure 3 shows carbon concentration away from GB carbides for several APT specimens extracted at different known depths for the HT-9 specimen irradiated to 280 dpa. For specimens free of any GB carbides, each data point was computed based on the mass spectrum of 20 nm wide slices along the length of the APT specimen. Similar measurements were not made for the APT specimen taken from a depth of 500–600 nm because the GB carbides could not be excluded from the slices. The dashed lines show the average carbon concentration computed for a large portion of an entire APT specimen that excludes GB carbides. The carbon concentration is higher near the ion irradiated specimen surface (e.g. ~3 at% at depth ~300 nm) and falls off deeper in the material (e.g., ~2 at% at depth ~1000 nm), matching the trend observed in the SIMS data provided in Fig. 1. Such high levels of carbon beyond the 0.8 at% nominal level for this material can only be attributed to carbon contamination during ion irradiation. The lack of dissolution of GB carbides, as evidenced by one of the APT specimens from the 280 dpa irradiated HT-9 specimen, offers evidence that the carbon came from an *ex-situ* source and further supports this determination. The carbon-rich matrix clusters were observed at all depths, and they are similar to those observed in the HT-9 irradiated to 100 dpa. The number density and size of the matrix carbon-rich clusters in the ion irradiated HT-9 are listed in Table 2. On average, these carbon clusters are about 3–4 nm in diameter with a very high number density. The APT specimen from a depth of 500–600 nm has similar carbon cluster size and number density to the other APT specimens, again suggesting that dissolution of preexisting GB carbides did not contribute to the formation of these small clusters.

**Table 2:** Carbon cluster statistics measured in ion irradiated materials. All clusters were defined by 6.8 at% isoconcentration surfaces.

Specimen	Depth (nm)	C entire tip (at%)	No. Density ( × 10^23^/m^3^)	Diameter and St. Dev. (nm)
HT-9, 100 dpa 400 °C	0–60	1.10	2.98	3.27 ± 1.68
HT-9, 280 dpa 400 °C	300–440	3.33	5.53	4.38 ± 2.46
	500–600	2.97†	3.43	3.63 ± 1.96
	700–800	2.92	3.90	3.57 ± 2.01
	1000–1250	2.03	1.83	3.43 ± 1.94

^†^GB carbides are excluded from this measurement.

Depth-specific concentrations of other elements are also shown in Table 1. The specimen taken from a depth of 500–600 nm had two GB carbides that were excluded from the composition measurement. The GB carbides appeared to have locally depleted the Cr content in the matrix but otherwise had no obvious effect on composition. The depth profile of the major alloying element, Cr, is not strongly affected by the small carbides. Cr was lower at a depth of 1000–1250 nm due to injected Fe. Other minor alloying elements, such as Si, Mn, W, V, Mo and Ni are very similar across all APT specimens, except at 1000–1250 nm their concentration slightly decreased due to injected Fe coming to rest in this region.

**Figure 4:**
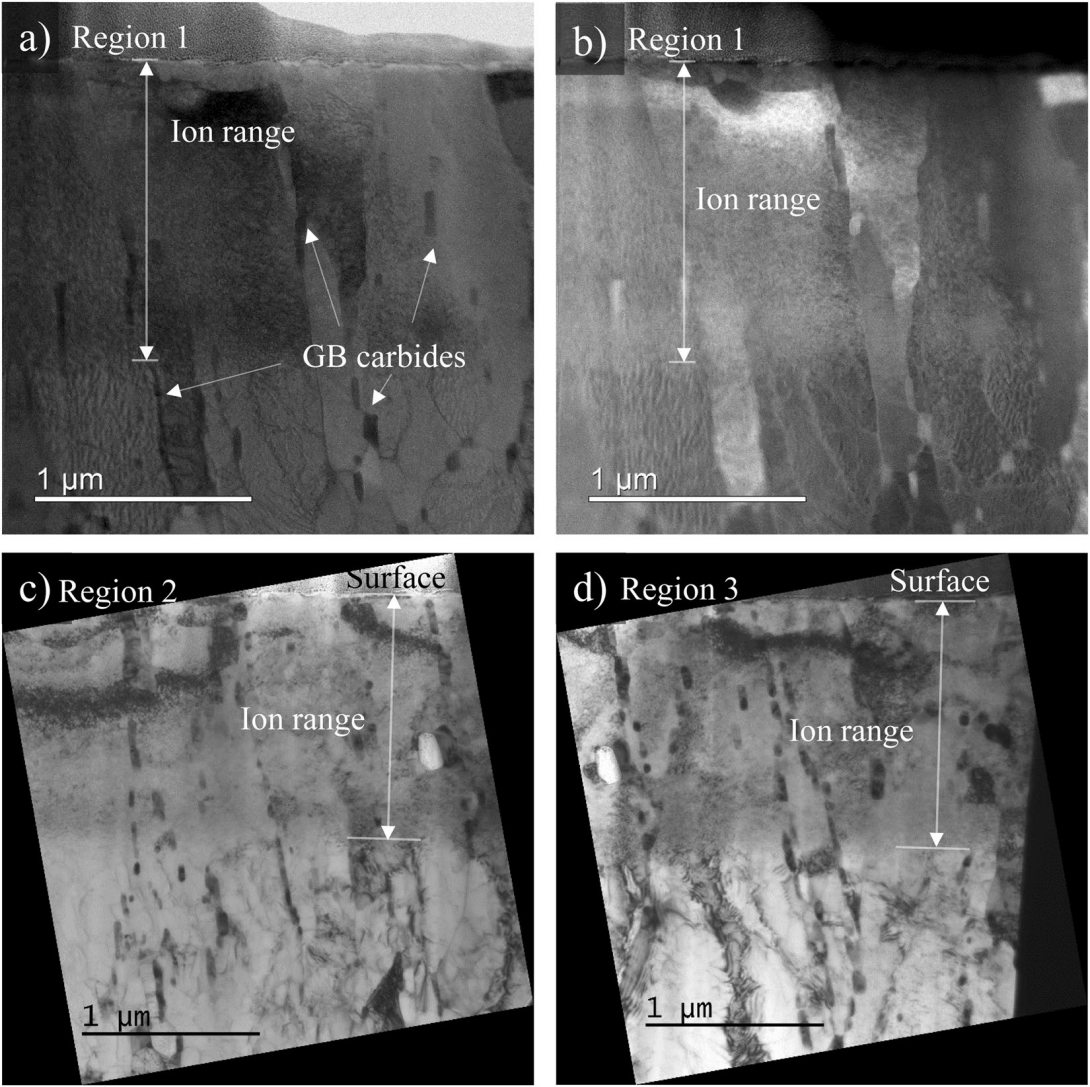
General microstructure for irradiated and unirradiated regions of ion irradiated HT-9 at three different locations. (**a**) STEM bright field (BF) and (**b**) STEM high angle annular dark field (HAADF) images of the same region; (**c**) and (**d**) TEM BF of another two different regions.

The general microstructure of HT-9 ion irradiated to 280 dpa was characterized by TEM at three different regions, and the result is shown in Fig. 4. Both scanning TEM (STEM) and TEM images suggest that grain/lath structures are well maintained in the irradiated region when compared with the unirradiated region beyond the ion range. Carbides, likely M_23_C_6_, in the size of tens to hundreds of nanometers were observed on grain boundaries in both the irradiated and unirradiated regions with no obvious difference in size or distribution between the two regions.

**Figure 5:**
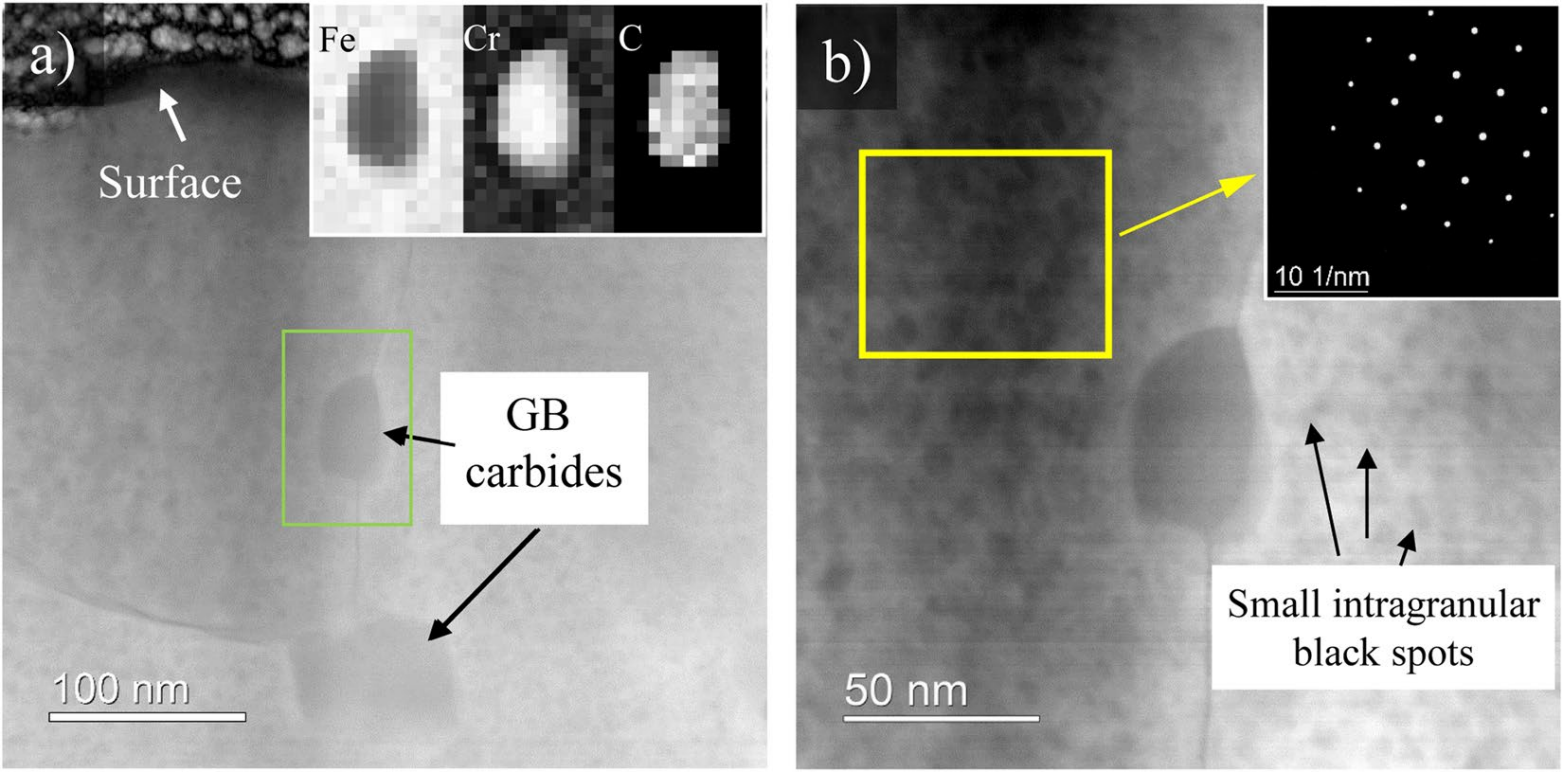
STEM HAADF images showing the intergranular carbides in both (**a)** and (**b**). The inset in (**a**) is the EELS map from the region around a GB carbide. The inset in (**b**) is the diffraction pattern that was acquired for the matrix region so that interference of GB carbides was excluded. The small intragranular dark spots in the STEM HAADF images are likely due to atomic number contrast from low atomic number precipitates.

Higher magnification STEM HAADF images of the irradiated region in HT-9 are shown in Fig. 5. Two GB carbides about ~50 and ~100 nm in diameter can be clearly seen near the incident irradiated surface. Elemental mapping using electron energy loss spectroscopy (EELS) shown inset on top right of Fig. 5a reveals that the GB carbides are enriched with Cr and C, and depleted in Fe. The carbon concentration in the GB carbide based on EELS spectrum quantification was determined to be ~20 at%, in agreement with M_23_C_6_ stoichiometry. The small intragranular dark spots in the STEM HAADF images (Fig. 5) could be due to second phases that contain low atomic weight elements such as carbon. As shown in the <100> zone axis diffraction pattern of a matrix region in Fig. 5b, only the Fe matrix was observed without any indication of a second phase. This suggests that the clusters are not crystallographically unique from the matrix. One possibility is α′ formation. 400 °C is within the temperature range for the α′ phase formation during irradiation of 12Cr F/M steels, and free carbon may segregate to the α′ phase due to the strong chemical affinity between Cr and C. A similar phenomenon has been previously reported in delta ferrite grains during carburization of duplex stainless steels^21^. The authors suggested that the spinodal-like decomposition into α-α′ may be assisted by carbon.

**Figure 6:**
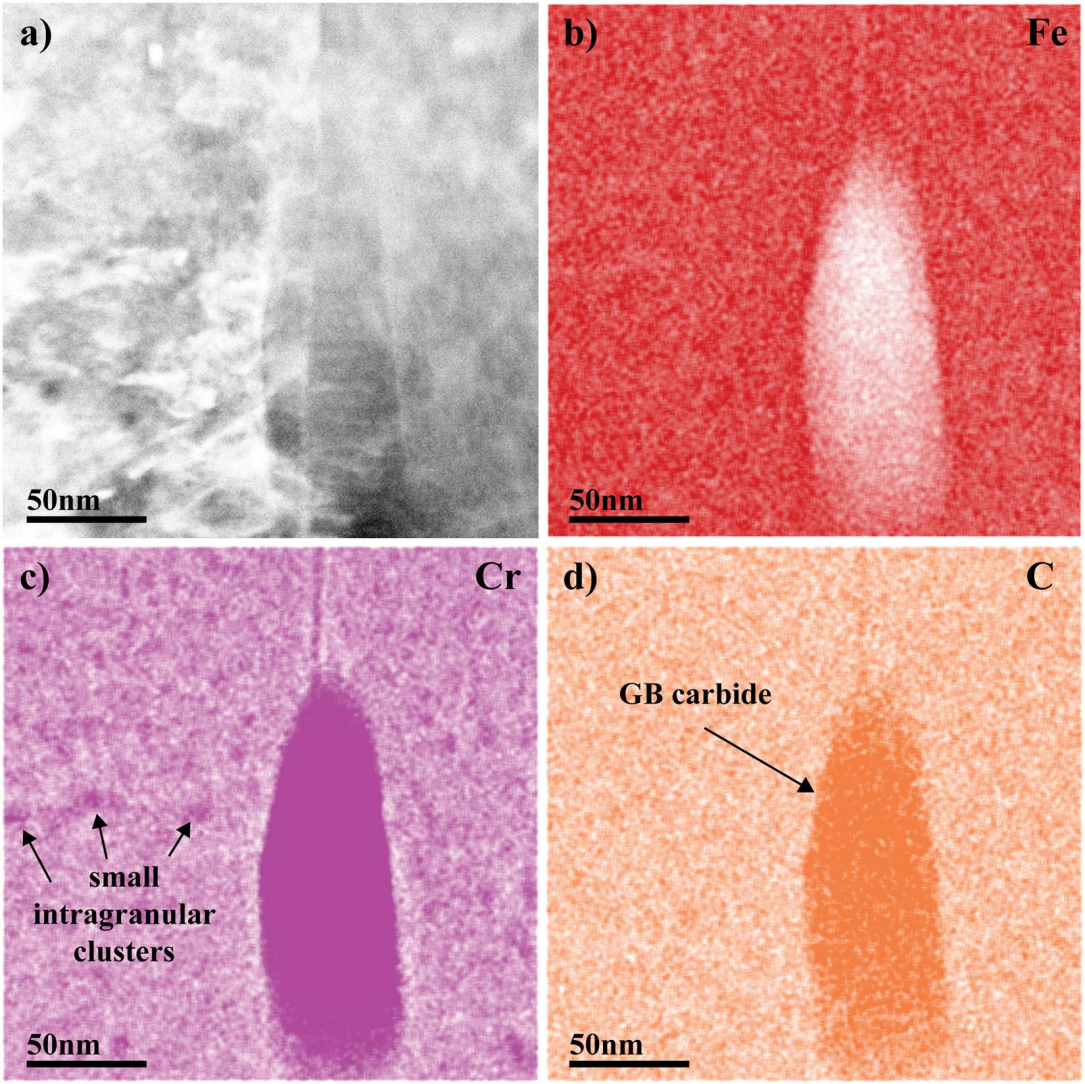
(**a**) STEM HAADF image near a grain boundary; (**b**), (**c**), and (**d**) high magnification EDS maps near peak damage showing small Cr segregation.

Energy dispersive spectroscopy (EDS) maps acquired at high magnification near the peak damage region are shown in Fig. 6. The Cr map shows that small (5–10 nm) Cr-rich clusters are likely present inside the grains. Because carbon emits a weak 282 eV x-ray and has a small interaction crosssection, it is difficult to map its presence in carbides that are much smaller than the specimen thickness, such as for the matrix carbide clusters here. Unfortunately, a statistically meaningful C signal could not be measured even after long (30 min) counting times. Thus, quantification of the Cr and C in these localized precipitates was not possible by EDS because the small volume fraction of these precipitates produces a weak signal compared to the matrix signal.

**Figure 7:**
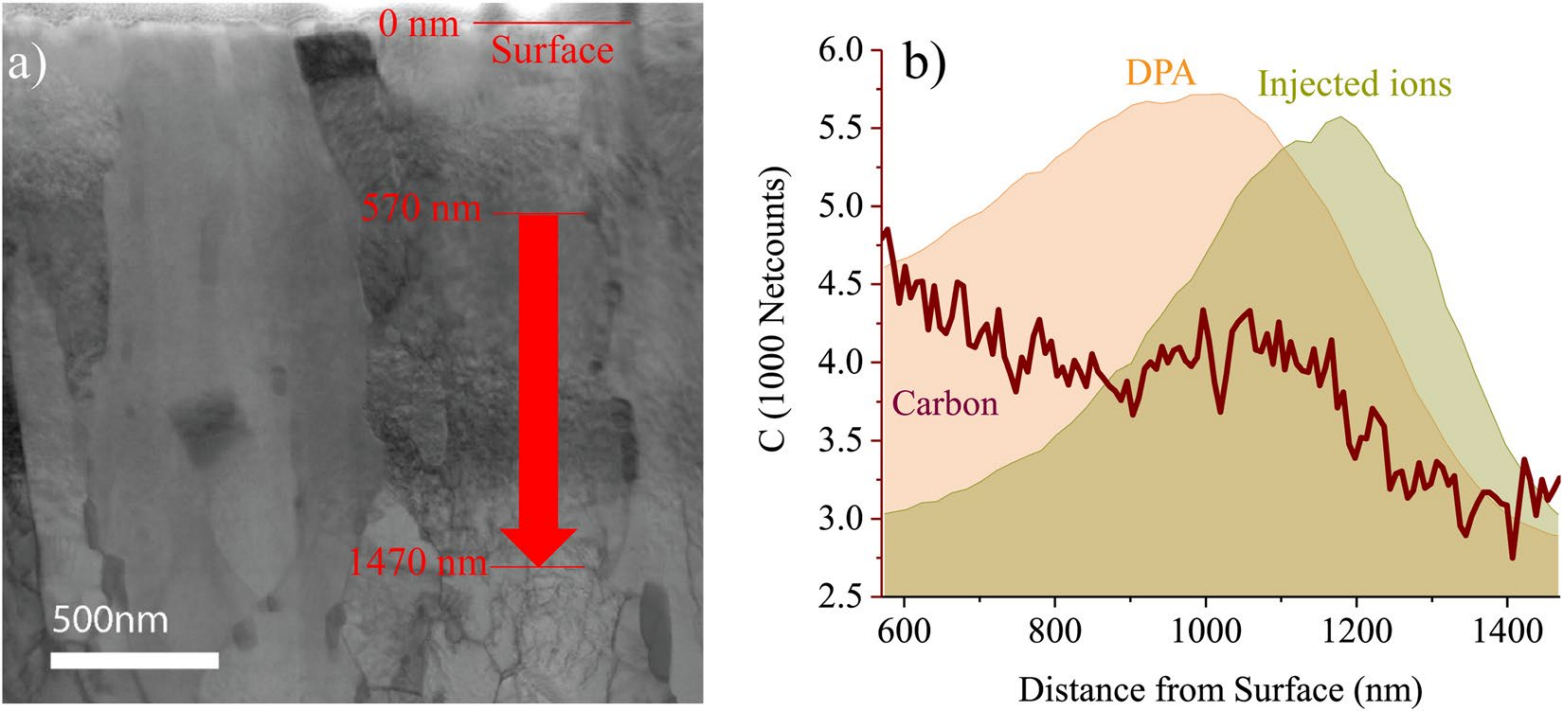
Line profile of carbon concentration from irradiated region to unirradiated region in 280 dpa HT-9. The line profile was obtained in a box scan with width of ~125 nm.

While detecting the quantity of carbon in small carbides that are dispersed within the matrix is a challenge, C in carbides and in solution near the surface of a TEM specimen will still emit x-rays that can be used to assess carbon content by EDS. The EDS raw signal was processed using an option called “netcounts” in ThermoFisher Scientific's Pathfinder X-ray Microanalysis software. It determines the integrated x-ray peak counts after background subtraction. The contribution of the Bremsstrahlung effect as electrons are decelerated when they interact with the field of the nuclei in the specimen is also removed during processing. The netcounts map is a qualitative, relative measurement that doesn’t remove thickness effects; hence, a region of constant thickness without substantial bending is desired. The relative abundance of carbon between irradiated and unirradiated regions as measured by netcounts is shown in Fig. 7b. The line scan region shown in Fig. 7a was selected in a region free of visible GB carbides. The carbon “netcounts” signal detected in the irradiated region is noticeably higher than that observed in the unirradiated regions. This result suggests that there is more carbon in the irradiated region. When combined with quantitative observations of changes in the preexisting GB carbide microstructure, this method may serve as a reasonable tool for assessing the possibility of carbon contamination by TEM.

## Discussion

Our measurements and observations suggest that SIMS depth profiling is undoubtedly the fastest and easiest technique described here to detect carbon contamination in ion irradiated F/M steels. This approach requires no additional sample preparation since the specimen surface is polished prior to ion irradiation. A qualitative measurement by depth profiling starting from the surface and ending well into the unirradiated region would be sufficient to determine the presence of carbon contamination. By using the unirradiated region as a standard for carbon concentration, the average carbon concentration can be obtained as a function of depth. The total carbon contamination level in the irradiated region can then be estimated. Limited microstructural information can be provided by SIMS depth profiling analysis, so if the results indicate significant ingress of carbon (and/or possibly other elements, such as oxygen and nitrogen), then further analysis using APT and/or TEM/STEM may be needed to understand the impact.

Although APT is quite sensitive to all chemical elements, the small analysis volume makes it difficult to determine whether the observations are representative of the bulk. Usually several APT specimens from different locations are required to obtain a full picture of the microstructure. For example, in this study where HT-9 was irradiated to 100 dpa, although APT successfully detected the carbon concentration to be ~1.1 at%, which is higher than nominal value of 0.8 at%, it is still difficult to confirm that such elevation is due to contamination rather than the dissolution of preexisting GB carbides. Other complementary techniques, either SEM or TEM, are needed to confirm the fate of those carbides. The time and effort would be much greater than applying ToF-SIMS. It is suggested that detecting carbon contamination by APT alone with high certainty is only possible when it is well beyond the bulk carbon level of the unirradiated material so that dissolution of pre-existing GB carbides could not explain the elevated matrix carbon/carbide levels. For example, in HT-9 the contamination needs to perhaps be ≥1.5x the unirradiated bulk carbon level to easily conclude the presence of contamination by APT.

Similar to APT, accurate detection of carbon contamination in F/M steels by TEM requires multiple types of observations. This can be attributed to several factors. First, EDS is only weakly sensitive to the carbon signal due to the small interaction crosssection and the absorption of the weak 282 eV x-ray. This issue was amplified by the carbon segregating to form intragranular Cr and C rich clusters at the nanometer scale. Detecting such carbon clusters in EDS maps is extremely challenging, if not impossible. An EDS line scan across the irradiated region and into the unirradiated region in an area without GB carbides did successfully pick up an elevated carbon level in the matrix of the irradiated region as compared to the unirradiated region, but quantification of the GB carbide population in the irradiated material would be needed to determine if the EDS signal represented contamination. Common imaging modes fail to detect those clusters as well. One technique not applied to identify C clusters in this study was STEM-EELS. While it is generally considered to be more suitable for detection of carbon as compared to EDS, it still has limited capability to quantify carbon levels in small C clusters in these materials and only provides for the possibility of improved elemental-based visualization.

The ability to detect carbon contamination by TEM may vary with irradiation temperature. Neutron irradiation at 400 °C typically does not cause matrix carbide formation in HT-9 and presumably would not lead to carbide formation during self-ion irradiation without contamination, so the presence of these small carbon clusters with no obviously unique crystal structure is a good indicator of possible contamination. However at higher irradiation temperatures where matrix carbides do form in HT-9^22^, detection of carbon contamination via the presence of a matrix second phase may be more challenging by TEM because there may be very little obvious difference in the microstructure.

One observation previously mentioned is that the matrix carbon enrichment in our specimens appears to be in the form of C enriched α. The only carbides having a crystal structure unique from the matrix were the preexisting M_23_C_6_ carbides on the grain boundaries. This is quite different from observations of matrix M_3_C and M_7_C_3_ carbides caused by carbon contamination in a recent work by Gigax *et al*.^23^ where ion irradiations were performed at 450 °C. It is likely the small increase in irradiation temperature for their study is causing the change in the types of carbides that are formed. In particular, α formation is known to occur in HT-9 at irradiation temperatures below 450 °C. A planned series of experiments would be needed to confirm the reasons for the difference in matrix precipitates for the two studies.

Using ToF-SIMS, carbon contamination in ion irradiated HT-9 can be quantitatively estimated by integration of the carbon concentration profile followed by subtraction of the baseline carbon level of ~0.8 at%. The results of such calculations are listed in Table 3 for the two irradiated specimens. The average carbon contamination levels in the irradiated region increase from ~0.29 at% in 100 dpa specimen to ~2.30 at% in 280 dpa specimen, corresponding to carbon contamination levels of 36% and 287% compared with initial carbon content.

**Table 3:** Carbon contamination quantification by ToF-SIMS.

Materials	Fluence (ions/cm^2^)	Carbon Contamination, at% (average)
HT-9 100 dpa 400 °C	1 × 10^17^	0.29
HT-9 280 dpa 400 °C	2.8 × 10^17^	2.30

APT analysis is able to readily quantify carbon concentration. Since the carbon concentration within grains of the unirradiated F/M steel is extremely low, and the GB carbides were observed by TEM to be stable under ion irradiation, the carbon concentration measured within grains by APT can be used as an estimate of the carbon contamination level. Examples of this type of measurement were provided in Table 2. The total carbon contamination level can then be estimated by interpolating values in APT datasets from different depths. Average carbon contamination values by this method were found to be ~2.08 at% for the 280 dpa specimen where APT specimens were extracted at several different depths.

Accurate measurement of carbon concentration in steels by APT needs special attention and experimental condition should be selected carefully. Takahashi *et al*.^24^ and Miyamoto *et al*.^25^ reported that under certain acquisition conditions, the measured carbon concentration could be higher than the real carbon concentration in cementite and Fe-C binary alloys because of partial loss of Fe ions due to the detector’s limitation to identify multiple hit events of the same iron isotopic ions. Their APT experiments were conducted in voltage-pulsing mode at a temperature of 20–90 K and demonstrated that the carbon concentration eventually converged to expected nominal values at the high end of the specimen temperature range during data collection. A similar study on cementite conducted by Kitaguchi *et al*.^26^ reported that although variations of carbon concentration with respect to acquisition parameters were observed, all measurement results were close to the real concentration in laser pulsing mode. All our APT data acquisitions were carried out in the laser mode in a moderate temperature range, and thus the APT-measured carbon concentration is expected to be of good accuracy.

Another difficulty is the overlap of some ion species in the mass spectrum. During APT operation, carbon in F/M steels can form complex molecular ions such as C_2_^+^, C_2_^++^, C_3_^+^, C_3_^++^, etc., besides commonly tabulated C^+^ and C^++ ^^26^. These complex ions overlap with some other species and make simple composition analysis less accurate. To accurately quantify composition, peak decomposition assisted by isotopic ratios should be used for mass spectrum-based analysis to account for ion species whose mass-to-charge ratios overlap with those of other species. In addition, the small analysis volume may not be representative if carbon contamination leads to the formation of large but lower number density precipitates that are not entirely inside an APT specimen.

Unlike APT, measuring carbon concentration using TEM has always been challenging. One possible way to estimate carbon content is to quantify the size and density of carbides in the matrix. Often the carbide-type can be reliably identified with electron diffraction techniques. This information can be used to obtain an estimate of the C concentration, but ignores almost all carbon atoms in solution. This method is not applicable to the current TEM data because accurate measurements of carbide size, number density, and crystal structure were not possible. EDS or EELS based techniques can also be applied to quantify carbon concentration in carbides; however, determination of carbon in the solution is also challenging.

There are a number of ways that carbon contamination could affect microstructural development during ion irradiation. Based on observations in this study, a higher carbon concentration may affect α′ formation, or as described in reference^23^, a greater amount of carbon contamination can cause the formation of a higher density of carbides. A high density of carbides or carbon-rich clusters caused by carbon contamination may serve as additional defect sinks in the material. As a result, point defect concentrations and interactions within the microstructure may be perturbed. This may result in the deviation of microstructural evolution from scenarios where there is no contamination.

Carbon interstitials may also affect microstructural evolution due to strong interactions with point defects or defect clusters. Direct evidence about the formation vacancy and interstitial carbon binding in α-Fe by positron-lifetime measurements were reported in the literature^27,28^. Since then, many theoretical modeling investigations have been conducted on carbon-point defect interaction in the BCC Fe system. The exact value of the binding energy is still under discussion, but almost all studies agree a strong interaction exists between carbon and vacancies and vacancy clusters^29–33^. The thermal stability of potential carbon-vacancy complexes have been studied by Terentyev *et al*., and the result suggests that within our experimental temperature range, some types of those complexes are still stable^34^. Simulation suggests that the carbon-vacancy interaction may also affect lattice damage recovery^35^. Carbon interstitials have been considered to affect self-interstitial atom (SIA) clusters and affect dislocation loop evolution in the BCC Fe structure^33,36–39^. Abe *et al*., reported that carbon-vacancy complexes could interact with SIA loops and impact their one-dimensional glide^38^. A recent study has found that carbon contamination can effectively reduce void swelling in HT-9^23^. These observations suggest that significant carbon contamination might alter the microstructural evolution of ion irradiated ferritic/martensitic steels, so it is important to know if carbon contamination is present.

Other than disturbing microstructural evolution, carbon contamination may interfere with property measurements as well. For example, much higher nano-hardness may be measured, not only due to the carbon clusters or precipitates inhibiting dislocation motion, but also due to the supersaturated carbon interstitials that provide additional solution strengthening to the material.

In summary, this paper provides practical perspectives and suggestions on using ToF-SIMS, APT, and TEM to quantify carbon contamination induced by ion irradiation and characterizing the related microstructure. Significant carbon contamination will likely alter the microstructure evolutionary path of ion irradiated F/M steels, so it is important to know if it is present. Our results suggest that the fastest and easiest method to identify and quantify carbon contamination is to use SIMS to obtain a depth profile of carbon from the incident surface to a depth well into the unirradiated region. If there is significant carbon contamination, APT and TEM can also detect carbon contamination. However, common imaging modes or EDS mapping may not find carbon clusters for some ion irradiation conditions like the one in this study. EDS line scans were needed to discover carbon contamination. SIMS appears to be the most reliable and easiest way to determine carbon contamination, even to very low levels. Thus, we suggest that a routine check using ToF-SIMS maybe helpful to ensure the ion irradiation was free of contamination or was at an insignificantly low level. As noted in this study and in a related study^11^, it should be recognized that even in absence of any carbon contamination, the carbon profile versus depth in F/M steels is altered by ion irradiation. Techniques to limit the amount of carbon contamination in ion irradiations of iron based alloys are of vital importance to avoid potential adverse effects from carbon contamination.

To quantify carbon contamination in the BCC Fe-based system during ion irradiation, we suggest the following: 1. Perform depthprofiling using SIMS to probe possible contamination elements; 2. If information about the impact of contamination on microstructure is desired, perform APT and TEM analysis along with SIMS; 3. APT specimens from various depths can be prepared to cover the irradiated region and be compared with unirradiated material. Use laserpulsing at a common, well-accepted evaporation temperature (40–50 K) and moderate laser energy to collect data, or use voltage-pulsing mode at higher temperatures. Carbon complex ions (sometimes metal-carbon ions) should be accounted for in mass spectrum analysis, and peak deconvolution is preferred for composition analysis; 4. Multiple TEM imaging modes should be tested; EDS and EELS mapping both at high magnification and line scanning may be most effective.

## Materials and Experimental

### Materials and Ion Irradiation

HT-9 heat 91353 was used in this study. It is a prototypical F/M steel for current reactor material needs and has been routinely used as a baseline reference for investigating radiation damage tolerance. It is a commercial alloy that has demonstrated excellent swelling resistance for fuel cladding and duct in fast reactors^40–42^. The HT-9 for this study was normalized at 1038 °C for 30 min followed by air cooling to room temperature, and it was then tempered at 760 °C for 30 min followed by air cooling. The composition is listed in Table 4. The carbon concentration in this heat of HT-9 is about 0.8 at%, with the majority of carbon partitioned to GB carbides after normalizing and tempering. Unless stated otherwise, carbon levels will be reported in at%.

**Table 4:** Composition (wt%) of HT-9 for this study.

Specimen	Heat	Fe	Cr	C	Si	Mn	W	V	Mo	Ni
HT-9	91353	Bal.	11.8	0.17 (0.8 at%)	0.28	0.54	0.52	0.24	0.94	0.57

All materials were irradiated by 3.5 MeV Fe^++^ ions using a 1.7 MV Tandetron accelerator in defocused beam mode^43^. The beam current was controlled to ~90 nA producing a peak damage rate of 1.8 × 10^–3^ dpa/s. The dpa level was calculated by SRIM^44^ in Kinchin-Pease mode following a procedure suggested by Stoller *et al*.^45^. Surface sputtering and ion injection at high dose can potentially alter the SRIM-calculated result, especially at low ion energies^8^. However, these effects are minimal for 3.5 MeV Fe ions to our target dose^8^. The target chamber vacuum was maintained to 5 × 10^–7^ torr during ion irradiation. Specimens were fixed on a hot stage sample holder using carbon-free silver paste. A resistive heater on the hot stage was controlled by a variable transformer, and the irradiation temperature was closely monitored by a type K thermocouple on the edge of the stage. An auxiliary system using an infrared camera was also used to measure specimen temperature. The beam heating effect is small for this class of material because of its relatively high thermal conductivity. Two ion irradiations were carried out. One HT-9 specimen was irradiated to 100 dpa at 400 °C, and the other was irradiated to 280 dpa at 400 °C. Unless otherwise noted, dpa values are at the peak damage depth (~1000 nm for these accelerator conditions).

### Time-of-Flight Secondary Ion Mass Spectroscopy (ToF-SIMS)

Depth profiles of carbon were collected from the specimens using a ToF-SIMS spectrometer (TOF.SIMS5, IONTOF GmbH, Münster, Germany) operated in negative ion mode that is more sensitive to carbon. A dual beam depth profiling strategy was used. A 2.0 keV Cs^+^ beam was used as a sputter beam that enhances the yield of negative ions. The Cs^+^ beam was scanned on a 300 × 300 μm^2^ area, while a 25.0 keV Bi^+^ beam that was used for analysis was scanned on a 100 × 100 μm^2^ area at the center of the Cs^+^ sputter crater to collect data. The analysis area is large enough to cover hundreds of grains so that the measured chemical composition is representative of the bulk values. The crater depth was determined using a DEKTAK 150 stylus profilometer with 5 µm radius stylus and contact force of 10 mg. A constant sputter rate was assumed in data analysis. More ToF-SIMS experimental details can be found in previous publications^46,47^.

Although SIMS is generally considered a qualitative or semiquantitative technique, quantification is possible if standard samples are available. In this study, the unirradiated region of HT-9 can be used as a standard reference since the nominal carbon concentration was known. The relative strength of the carbon signal intensity between the irradiated and unirradiated regions can be used to estimate carbon concentrations. The concentration is usually considered to be correlated with the acquired signal counts in a nearly linear relationship, assuming that the secondary ion yield is almost a constant. This is the approach used in this study. More accurate quantification would require systematic measurement of the ion yields in standard specimens covering the range of carbon concentration in this study.

### Atom Probe Tomography (APT)

Atom probe tomography is a technique rapidly gaining popularity for characterizing microstructures of irradiated materials due to its unprecedented capability to obtain element-specific information at the nano-scale in 3D via tomographic data reconstructions^48,49^. APT specimens were fabricated using standard lift-out and milling procedures^50,51^ using an FEI 3D Quanta FEG Dual-Beam Focused Ion Beam (FIB). Depth-specific specimens can be made by closely monitoring and shaping the specimen geometry with a low energy Ga ion beam. APT needles were analyzed with a Cameca LEAP 4000X HR at the Environmental Molecular Sciences Laboratory (EMSL) at Pacific Northwest National Laboratory (PNNL). APT specimens were cooled to 40 K in the analysis chamber, and data collection was operated in laser-pulsing mode with a laser energy of 60 pJ, pulse rate of 200 kHz, and detection rate of 0.3%.

Data reconstruction and analysis were carried out in IVAS version 3.6.12. Carbon-rich precipitates were analyzed by constructing isoconcentration surfaces based on the signature element carbon. Isoconcentration surfaces are surfaces on which a designated element is of constant concentration as defined by user input^52^. The composition along the radial direction of precipitates, called a proximity histogram (proxigram), can also be calculated. In a proxigram plot, the isoconcentration surface is labeled as position zero.

### Transmission Electron Microscopy (TEM)

TEM cross-section lamella specimens were fabricated using standard FIB lift-out in an FEI Quanta. TEM and STEM analysis was performed in a spherical-aberration corrected JEM-ARM200CF TEM/STEM microscope operated at 200 kV equipped with a Gatan GIF Quantum energy filter and a Centurio silicon drift detector for EDS. Microstructures of irradiated material, such as grain structure and dislocation and precipitate distributions, were characterized using (S)TEM. Elemental analysis was performed with EDS and EELS. The electron beam current for EDS analysis was fixed at 15 μA. The analysis of EDS results was performed using Pathfinder X-ray microanalysis software from ThermoFisher Scientific. EELS quantification was conducted in a Gatan GMS3. A power law background correction was performed for each edge, and the edges were fitted with the Hatree-Slater model. The convergence and collection angles for EELS analysis were 27.5 mrad and 82.6 mrad, respectively.

### Data availability

The datasets generated during and/or analyzed during the current study are available from the corresponding author on reasonable request.
